# Adverse drug reaction signal detection via the long short-term memory model

**DOI:** 10.3389/fphar.2025.1554650

**Published:** 2025-06-23

**Authors:** Mengqi Cao, Yanna Chi, Jinyang Yu, Yu Yang, Ruogu Meng, Jinzhu Jia

**Affiliations:** ^1^ Department of Biostatistics, School of Public Health, Peking University, Beijing, China; ^2^ Department of Genetics, Peking University Cancer Hospital and Institute, Beijing, China; ^3^ Center for ADR Monitoring of Guangdong, Guangzhou, China; ^4^ National Institute of Health Data Science, Peking University, Beijing, China; ^5^ Center for Statistical Science, Peking University, Beijing, China

**Keywords:** adverse drug reaction, signal detection, free text, the long short-term memory model, feature importance

## Abstract

**Introduction:**

Drug safety has increasingly become a serious public health problem that threatens health and damages social economy. The common detection methods have the problem of high false positive rate. This study aims to introduce deep learning models into the adverse drug reaction (ADR) signal detection and compare different methods.

**Methods:**

The data are based on adverse events collected by Center for ADR Monitoring of Guangdong. Traditional statistical methods were used for data preliminary screening. We transformed data into free text, extracted text information and made classification prediction by using the Long Short-Term Memory (LSTM) model. We compared it with the existing signal detection methods, including Logistic Regression, Random Forest, K-NearestNeighbor, and Multilayer Perceptron. The feature importance of the included variables was analyzed.

**Results:**

A total of 2,376 ADR signals were identified between January 2018 and December 2019, comprising 448 positive signals and 1,928 negative signals. The sensitivity of the LSTM model based on free text reached 95.16%, and the F1-score was 0.9706. The sensitivity of Logistic Regression model based on feature variables was 86.83%, and the F1-score was 0.9063. The classification results of our model demonstrate superior sensitivity and F1-score compared to traditional methods. Several important variables “Reasons for taking medication”, “Serious ADR scenario 4”, “Adverse reaction analysis 5”, and “Dosage” had an important influence on the result.

**Conclusion:**

The application of deep learning models shows potential to improve the detection performance in ADR monitoring.

## Introduction

Drugs may cause harmful and unexpected adverse reactions at normal dosages, known as adverse drug reaction (ADR) (Edwards and Aronson, 2000). Drug safety imposes significant public health and socioeconomic burdens. Severe ADRs can worsen patients’ outcomes and even lead to mortality. Death due to ADRs has become the fifth most common cause of in-hospital mortality (Nguyen et al., 2021; Krähenbühl, 2015). The review showed that worldwide, ADRs impose an annual cost of $ 75 billion on healthcare systems (Margraff and Bertram, 2014). Under-reporting is its major limitation drug safety monitoring (Lopez-Gonzalez et al., 2009). Consequently, how to effectively identify and predict ADRs, prevent them, and improve drug safety is currently a research focus (Al Meslamani, 2023).

Traditional approaches to drug safety regulation mainly include spontaneous reporting systems, such as VigiBase (the World Health Organization’s global database of individual case safety reports), and the National Adverse Drug Reaction Surveillance System of China (Hou et al., 2016). Currently, common ADR signal detection methods based on spontaneous reporting systems integrate the reporting odds ratio (ROR), proportional reporting ratio (PRR), the multi-item Gamma-Poisson Shrinker (MGPS), and the Bayesian Confidence Propagation Neural Network (BCPNN) (Northardt, 2024).

These detection methods generally have high false positive rates (Rothman et al., 2004). In addition, these methods utilize only a limited subset of variables from the self-reporting system, leading to insufficient data utilization (Jiao et al., 2024). Studies have demonstrated that incorporating additional information, such as the timing of ADR occurrences, can enhance detection specificity and identify signals undetectable by traditional methods (Jiao et al., 2024; Liu et al., 2019). Furthermore, traditional approaches require direct monitoring and organization of relevant information from large datasets, which demands significant time and human resources (Yamamoto et al., 2023).

The rapid development of deep learning provides a good solution to these shortcomings (Lee et al., 2024). The time-series processing model based on deep learning can identify and process variables related to occurrence timing to improve the monitoring and early warning capability of ADR signals (Gao et al., 2022; Munkhdalai et al., 2018). Research (Sarker and Gonzalez, 2015) indicate that using advanced natural language processing techniques for generating information rich features from text can significantly improve classification accuracies. Deep learning models can also incorporate more data and variables to maximize the use of information from the reporting system without requiring extensive manual effort. The application of deep learning models in healthcare highlights their public health significance (Al Meslamani, 2023).

## Materials and methods

### Data sources

ADR reports were collected by the Center for ADR Monitoring of Guangdong from 2018 to 2019. The 2018 dataset contained 143,406 reports, and the 2019 dataset contained 137,708 reports. Positive cases were manually validated by ADR experts through on-site investigations.

In 2018 dataset, 283 positive cases (0.197% of the total reports) were confirmed, while 165 positive cases (0.120% of the total reports) were confirmed in 2019 dataset. Negative cases for model training were selected using the PRR, ROR, BCPNN, and MGPS methods. The results of these methods were ranked by descending *p*-values. Negative cases were sampled at a ratio of 3–6 times the number of positive cases. Ultimately, 1,031 negative cases were selected from the 2018 dataset and 897 from the 2019 dataset. Data from 2018 to 2019 were merged into a combined dataset.

### Data preprocessing

Each signal in the dataset initially contained 72 feature variables. Variables not associated with ADR signals, recurring variables, and those with a missing data rate exceeding 20% were excluded (details of excluded variables are provided in Supplementary Table S1). After exclusions, 48 variables were retained for model calculations. The classification and inclusion criteria for these variables are summarized in Table 1.

**TABLE 1:** Inclusion and classification of variables.

Classification	Variable
Categorical Variable	Adverse Reaction Status, Unit type, Department, Gender, ethnicity, Family ADR, Past ADR, Adverse reaction result, Impact on primary disease, Adverse reaction occurrence abroad, Reporting evaluates, Reporting unit evaluates, the Municipal ADR monitoring institution, Adverse reaction analysis 1∼5, Serious ADR scenario 1∼7, Suspected concomitant drug, Trade name, Common name, Dosage form, Reasons for taking medication, Manufacturer, Batch number, Frequency, Usage, Drug classification, Pharmacological effect, Hospital level, Provincial bureau evaluation, Drug standards
Numerical Variable	Date of birth, Weight, Adverse reaction occurrence time, Start time, Dosage, Dosage unit
Describe or explain	ADR System, Adverse reaction event name, Primary disease

Prior to model application, the retained data underwent preprocessing. The variables included categorical, numerical, and descriptive types. Time-dependent variables were converted to numerical representations, while numerical variables retained their original values. Variables with a missing data rate below 20% or illogical values were filled according to the basic information of institutions and similar entries; unverifiable entries were replaced with “unknown”. Categorical variables were encoded via one-hot rule and the medical terms therein were combined into free text. Special symbols (e.g., punctuation, non-alphanumeric characters) were removed. Positive and negative cases were labeled as 1 and 0, respectively.

### Disproportionality analysis

Currently, disproportionality analysis is widely employed in post-marketing drug safety surveillance. This method assumes that if a drug is causally linked to specific adverse events (AEs), the observed frequency of the drug-AE combination will exceed its expected frequency, leading to an imbalance (Gosho et al., 2017). A potential safety signal is identified when this imbalance surpasses a predefined threshold.

Disproportionality analysis methods are categorized into frequency-based and Bayesian approaches (Northardt, 2024; Jiao et al., 2024). Frequency-based methods include the ROR and PRR. The ROR calculates the ratio of the observed frequency of the target drug-AE combination to the frequency of other drug-AE combinations. Similarly, the PRR compares the proportion of the target drug-AE combination with that of other drug-AE combinations. Bayesian methods include the BCPNN and MGPS. The BCPNN evaluates the strength of association between drugs and AEs using the information component (IC) and its confidence interval. In contrast, the MGPS employs the empirical Bayes geometric mean (EBGM) as its primary measure.

In this study, the ROR, PRR, BCPNN, and MGPS methods were applied to the dataset to identify ADR signals. The signal detection criteria for each method were defined as follows: $\left(1\right)\;ROR-1.96SE>1;\;\left(2\right)\;PRR-1.96SE>1;\;\left(3\right)\;IC-2SD>0;\;and\;\left(4\right)\;EB05>2.$ Additionally, the number of events of interest for the target drug ( $N_{11}$ ) was required to be ≥3 .

### Methods based on feature variables

Logistic regression (LR) is a statistical method used to estimate the probability of a binary outcome. It assumes a linear relationship between predictor variables (*independent variables*) and the log-odds of the target variable. The model converts these log-odds into probabilities via the logistic (*sigmoid*) function. Parameters are estimated using maximum likelihood estimation, which identifies coefficients that maximize the likelihood of the observed data.

Random Forest (RF) is an ensemble technique for classification and regression tasks. It constructs multiple decision trees during training, with outputs determined by majority voting (*classification*) or mean prediction (*regression*). To enhance diversity and reduce overfitting, the algorithm randomly selects subsets of data and features for each tree. At each split, a random subset of features is evaluated, and the optimal split is chosen using metrics such as *Gini* impurity or mean squared error reduction. Trees are grown fully without pruning to capture complex patterns. Predictions are aggregated across trees, improving accuracy and stability.

The k-nearest neighbors (KNN) algorithm is a straightforward yet effective method for classification in machine learning. It classifies new data points by comparing them to training instances using distance metrics (e.g., Euclidean distance). For an unseen instance, distances to all training points are computed, and the *k* nearest neighbors are identified (*k* is user-defined). The class label is assigned via majority vote or weighted vote, where closer neighbors have greater influence.

The Multilayer Perceptron (MLP), a feedforward neural network, is widely used for classification and regression. It comprises an input layer, hidden layers, and an output layer. Neurons in hidden and output layers apply activation functions to weighted inputs. During training, weights and biases are initialized randomly, and input data is processed through the network. Loss (e.g., cross-entropy for classification, mean squared error for regression) is calculated between predictions and targets. The backpropagation algorithm with gradient descent optimizes weights and biases iteratively to minimize loss. Once trained, the network generates predictions (class labels) by processing new data through its network.

### Model based on time-series and text processing

First, all feature variables for each entry were concatenated and converted into long free text as model input. The ‘occurrence time of adverse reactions’ was standardized to the *YYYY-MM-DD* format and incorporated as a time-series variable.

The Long Short-Term Memory (LSTM) network, a specialized recurrent neural network (RNN), addresses the gradient vanishing issue inherent in traditional RNNs (Yu et al., 2019). The LSTM architecture comprises memory cells regulated by input gates, forget gates, and output gates. These gates enable persistent information flow across time steps, allowing hidden states to retain long-term dependencies (Li and Yu, 2020). LSTMs are widely adopted for text and sequence modeling due to their capacity to capture long-range semantic patterns (Liu et al., 2022). In this study, LSTM model was employed to process ADR reports containing free-text narratives, medical terminology (Jagannatha and Yu, 2016), and time-dependent features (Santiso et al., 2019).

The LSTM equations are defined as:

$$i_{t}=\sigma \left(W_{ii}x_{t}+b_{ii}+W_{hi}h_{t-1}+b_{hi}\right)$$

$$f_{t}=\sigma \left(W_{if}x_{t}+b_{if}+W_{hf}h_{t-1}+b_{hf}\right)$$

$$o_{t}=\sigma \left(W_{io}x_{t}+b_{io}+W_{ho}h_{t-1}+b_{ho}\right)$$

$${\tilde{C}}_{t}=\tanh\left(W_{ic}x_{t}+b_{ic}+W_{hc}h_{t-1}+b_{hc}\right)$$

$$C_{t}=f_{t}⊙C_{t-1}+i_{t}⊙{\tilde{C}}_{t}$$

$$h_{t}=o_{t}⊙\tanh\left(C_{t}\right)$$

Where σ denotes the sigmoid activation function; $i_{t}$ , $f_{t}$ and $o_{t}$ represent input, forget, and output gate computations at time t ; $W_{*}$ and $b_{*}$ are weight matrices and bias vectors; $C_{t}$ is the cell state at time t ; ⊙ indicates the Hadamard product; and $h_{t}$ is the hidden state output at time t .

The dataset was partitioned into training, testing, and validation sets in a 7:2:1 ratio, with stratified sampling applied to maintain the balance between positive and negative entries across all subsets. Technical details of the training and hyperparameter tuning processes for the all models are provided in Supplementary Appendix S2. All model training processes were conducted on the Python 3.8 platform using version-compatible software packages.

### Feature importance

The chi-square value quantifies the discrepancy between observed and expected frequencies. A higher chi-square value for a feature variable indicates a greater discrepancy, reflecting stronger association between the variable and the outcome (Gosho et al., 2017). We used the chi-square value as a measure of feature importance to the outcome.

## Results

### Key results of the models

The LSTM model integrating time-series and text processing achieved a sensitivity of 95.16% and an F1-score of 0.9706, significantly outperforming comparative models in overall performance metrics. Among feature-based models, the LR model demonstrated the highest sensitivity (86.83%) and F1-score (0.9063), while the MLP exhibited the lowest sensitivity (54.57%) and F1-score (0.5494). Detailed performance metrics for all models are summarized in Table 2.

**TABLE 2:** Performance of LSTM and comparison models.

Models	Sensitivity	Precision	Accuracy	F1-score
LR	86.83%	94.78%	95.85%	0.9063
RF	83.06%	90.17%	94.01%	0.8647
KNN	70.43%	76.05%	88.45%	0.7313
MLP	54.57%	55.32%	86.64%	0.5494
LSTM	**95.16%**	99.04%	98.62%	**0.9706**

Bold values means the maximum value of the indicators.

**FIGURE 1:**
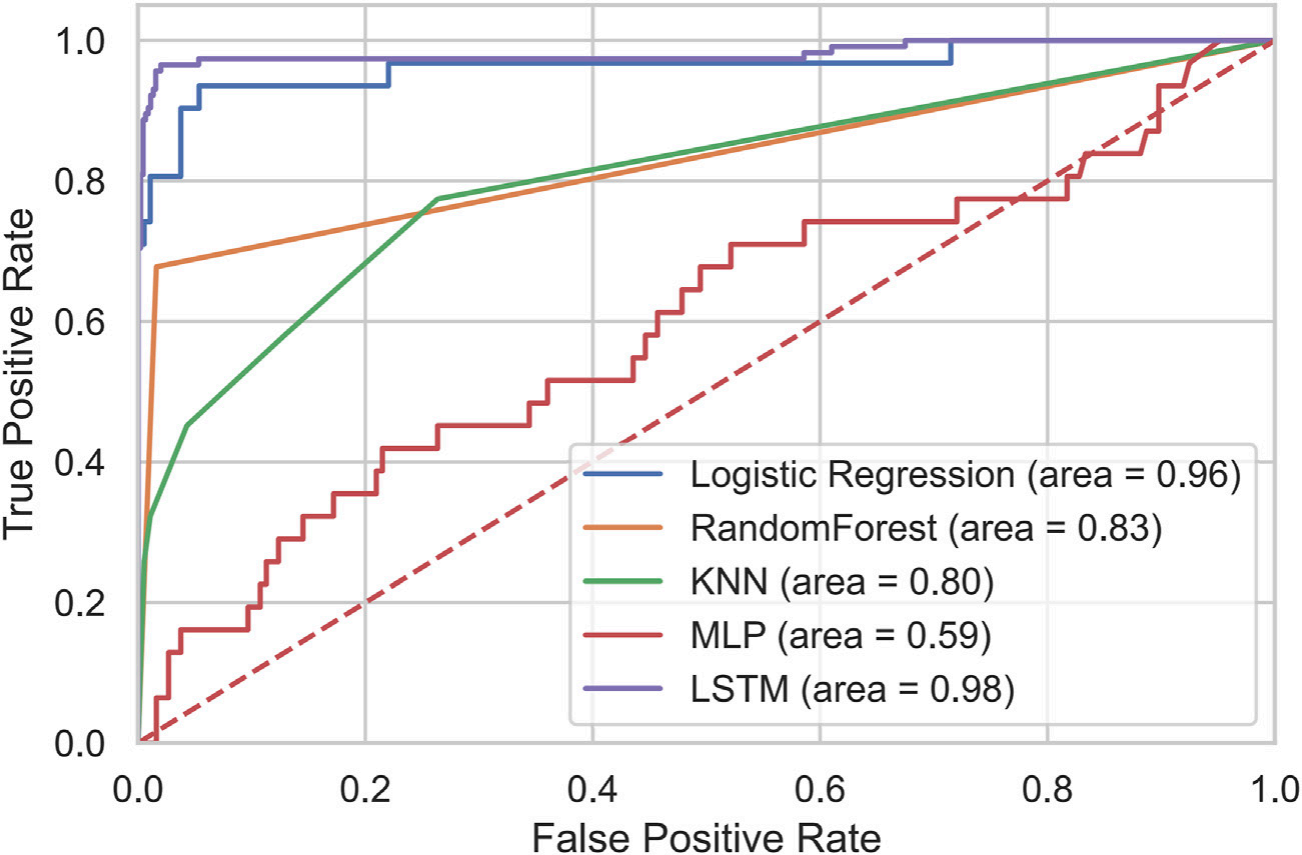
The ROC of LSTM and comparison models.

The receiver operating characteristic (ROC) curves and corresponding area under the curve (AUC) values for all models are shown in Figure 1. The LSTM model achieved the largest AUC (0.98), whereas the MLP model exhibited the lowest AUC (0.59).

### Results of disproportionality analysis

Among the disproportionality analysis methods, BCPNN achieved the highest sensitivity (73.80%), while MGPS showed the lowest sensitivity (44.92%). MGPS exhibited the highest specificity (84.82%), whereas PRR had the lowest specificity (58.46%). In terms of the composite metric Youden’s Index (YI), BCPNN yielded the highest value (0.3464), and PRR the lowest value (0.2691). In contrast, the LSTM model demonstrated a specificity of 96.31% with a notably low false positive rate (3.69%), resulting in a YI of 0.9147. Detailed performance metrics for these methods are summarized in Table 3.

**TABLE 3:** Performance of disproportionality analysis methods.

Methods	Sensitivity	False negative rate	Specificity	False positive rate	Youden’s Index^a^
ROR	68.45%	31.55%	58.62%	41.38%	0.2707
PRR	68.45%	31.55%	58.46%	41.54%	0.2691
BCPNN	73.80%	26.20%	60.84%	39.16%	0.3464
MGPS	44.92%	55.08%	84.82%	15.18%	0.2974
LSTM	95.16%	4.84%	96.31%	3.69%	0.9147

^a^ Youden’s Index = Sensitivity + Specificity-1.

### Feature importance score

**FIGURE 2:**
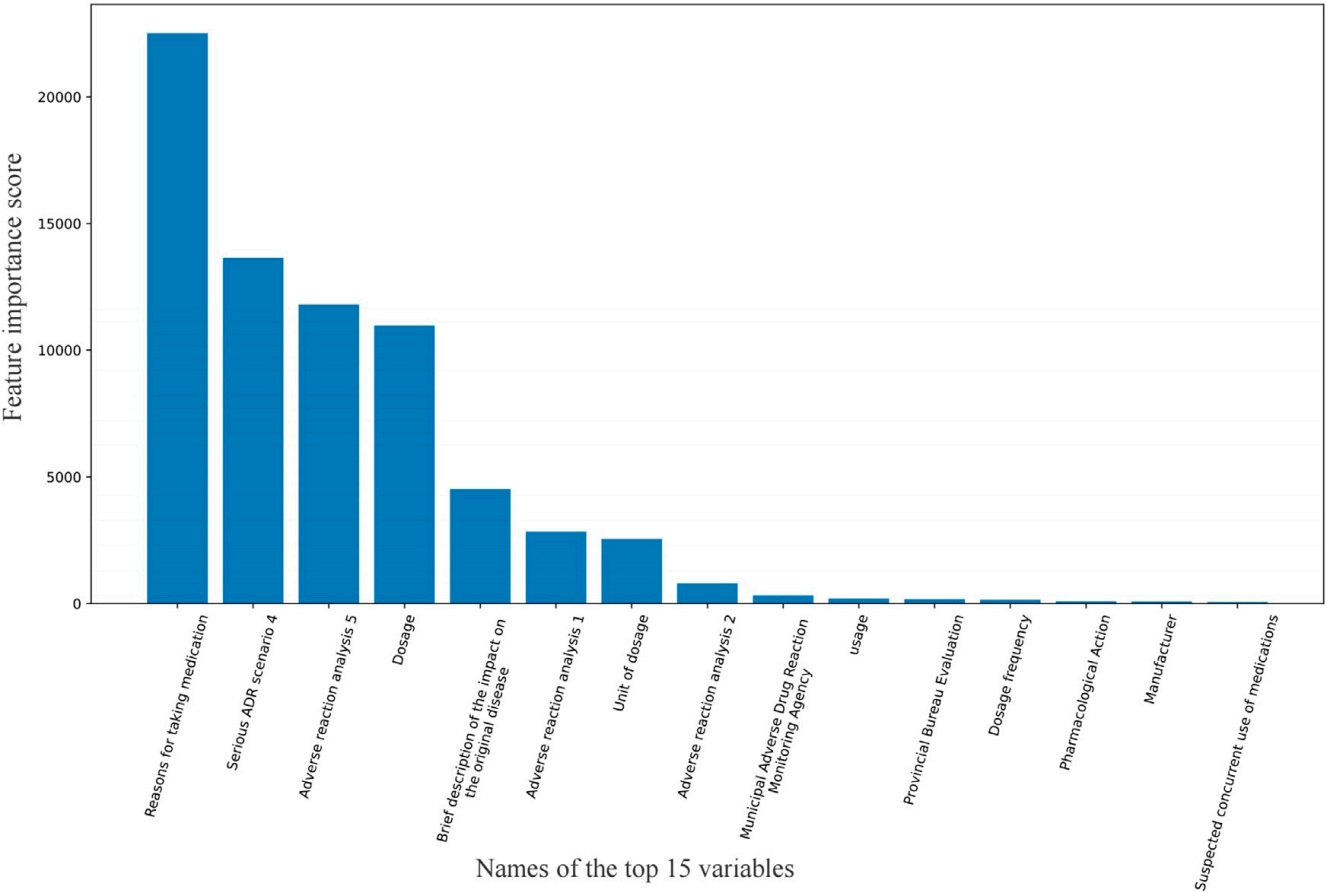
Feature importance score of variables.

The top 15 variables with the highest feature importance scores are shown in Figure 2. The highest-scoring variable was “Reasons for taking medication,” followed by “Serious ADR scenario 4,” “Adverse reaction analysis 5,” and “Dosage.” Here, “Serious ADR scenario 4” refers to *whether the adverse reaction caused permanent impairment of physiological functions*, while “Adverse reaction analysis five” indicates *whether the reaction resolved or diminished after drug discontinuation or dose reduction*.

## Discussion

This study explores the effectiveness of several different models for ADR signal detection. The results show that the LSTM model based on free text achieves the best predictive performance among the models evaluated. The LSTM model achieved superior predictive performance (sensitivity: 95.16%, F1-score: 0.9706) compared to traditional methods such as ROR, PRR, BCPNN, and MGPS, which are widely used in current monitoring systems but exhibit limitations in variable selection, bias control, and handling extreme values. While traditional methods remain useful for preliminary signal screening (as applied to negative data selection in this study), the LR method also performed robustly, likely due to its alignment with the proportional imbalance assumption inherent in disproportionality analyses (Sommer et al., 2024). However, other models (RF, KNN, MLP) showed suboptimal results, potentially due to class imbalance, high dimensionality, or overfitting risks (Carracedo-Reboredo et al., 2021; Deimazar and Sheikhtaheri, 2023). The This study also encourages us that implementation of innovative methods is essential to encourage ADR reporting (Joaquim et al., 2023).

The analysis highlights the critical role of specific variables, such as ‘Reasons for taking medication’, ‘Serious ADR scenario 4’, and ‘Adverse reaction analysis 5’, in signal detection. These findings underscore the importance of enhancing data quality through standardized reporting protocols and improved analytical capabilities at monitoring institutions. For instance, logical inconsistencies, missing data, and non-standardized entries—though partially addressed via technical imputation and variable exclusion—may still introduce bias, emphasizing the need for systematic data curation (Liu and Zhang, 2019). When reporting, some systems lack standardization, or data entry personnels need to fill in descriptive or explanatory language. Furthermore, class imbalance (predominance of negative samples) likely contributed to reduced sensitivity, as models tended to favor majority-class predictions (Arku et al., 2022).

Notably, the LSTM model’s insensitivity to sparse or irregular text inputs—a common issue in ADR reports—positions it as a scalable solution for processing free-text narratives (Edrees et al., 2022), especially with advancements in natural language processing. Nevertheless, several limitations must be acknowledged. First, the model was trained on data from a single province in China, limiting its generalizability to other regions or populations with divergent reporting practices or demographic profiles. Second, negative samples were selected using traditional disproportionality methods, which may exclude novel or atypical ADR patterns, introducing potential selection bias. Third, while technical imputation mitigated missing data, residual heterogeneity (e.g., inconsistent causality assessments) could affect model robustness (Yan et al., 2022).

To address these challenges, future work should prioritize multicenter validation across diverse healthcare systems, integration of active learning to capture atypical cases, and collaborative data sharing initiatives to enrich feature representation (Jiao et al., 2024). Specifically, incorporating data from diverse healthcare systems (including those within the same country and internationally) could assess the model’s generalizability to varied demographic and reporting practices, as demonstrated in recent multi-center pharmacovigilance studies (Hu et al., 2022; Saseedharan et al., 2024). Additionally, standardizing ADR reporting frameworks and raising public awareness of proactive reporting could further enhance data quality and model utility in public health (Liu and Zhang, 2019; Goldstein et al., 2013). The future path in drug safety solely depends on proactive pharmacovigilance approaches carried out by all stakeholders, where patients play a vital role in ADR reporting (Joaquim et al., 2023).

## Conclusion

This study demonstrates that deep learning models, particularly the LSTM network, significantly enhance adverse drug reaction (ADR) signal detection compared to traditional statistical and machine learning methods. Key variables emerged as pivotal predictors, underscoring the value of structured data fields and standardized reporting. These findings highlight the transformative potential of integrating natural language processing and deep learning into pharmacovigilance systems to improve drug safety monitoring.

## Data Availability

The raw data supporting the conclusions of this article will be made available by the authors upon request. Requests to access these datasets should be directed to Mengqi Cao, 18811332059@163.com.
