# Supplementary material for: Adverse drug reaction signal detection via the long short-term memory model
Source: Front Pharmacol. 2025 Jun 23;16:1554650. doi: 10.3389/fphar.2025.1554650 (PMC12230008; doi:10.3389/fphar.2025.1554650)
Supplement: Supplementary file 1 [file Supplementaryfile1.docx]

**Table S1**. Exclusion and classification of variables

| Classification | Variable |
| --- | --- |
| Irrelevant variable | Report number, Reporting unit, Telephone number, Patient contact information, Name of hospital, Cause of adverse reactions, City and district, Manufacturer's province, Provincial bureau evaluation description, Approval number |
| Recurring variables | Report date, Provincial center accepts time, Drug withdrawal time, Common name (A, B) |
| Variables with a missing rate exceeding 20% | Family adverse drug reactions details, Details of past adverse drug reactions, Time of death, Effect on the original disease (b, c), Brief description of the effects on the original disease, Brief description of similar adverse reactions abroad, Brief evaluation of municipal centers |

**Appendix S2:** Technical details of model training and hyperparameter tuning

**Logistic Regression (LR)**

Hyperparameter tuning for logistic regression was performed using **grid search** with **5-fold cross-validation**. Parameters were optimized via **maximum likelihood estimation (MLE)** with **class_weight='balanced'** (positive-to-negative class ratio 1:600) to address data imbalance. Regularization methods (**L1/L2**) were tested, and optimal penalty strength was selected through cross-validation. The default classification threshold was set to 0.5, with adjustments made to balance sensitivity and specificity.

**Random Forest (RF)**

Random Forest hyperparameters were tuned via **grid search** with **5-fold cross-validation**, exploring the following ranges: **n_estimators** from [100, 200, 300, 400, 500], **max_features** from [sqrt, log2, 0.5, 0.8], **max_depth** from [5, 10, 15, 20, None], **min_samples_split** from [2, 5, 10], and **min_samples_leaf** from [1, 2, 4]. Class weights were set to 1:600 to mitigate imbalance.

**k-Nearest Neighbors (KNN)**

For KNN, **grid search** over *k*values (1–30) and distance weighting schemes (**uniform** vs. **distance-based weights**) was conducted. Feature subsets of 10, 20, or 30 features were evaluated. Model robustness was validated using **5-fold cross-validation**.

**Multilayer Perceptron (MLP)**

The MLP architecture included an input layer (51 neurons), hidden layers (1–3 layers with 32–128 neurons per layer, **ReLU activation**), and an output layer (**sigmoid activation**). Hyperparameters were optimized via **grid search** over learning rates [0.001, 0.01, 0.1, 0.5], batch sizes [32, 64, 128], and L2 regularization coefficients [0.01, 0.001]. Training used the **AdamW** optimizer (initial learning rate 0.001) and **early stopping** (10 epochs without validation loss improvement).

**Long Short-Term Memory (LSTM)**

The LSTM network was implemented using a sequential architecture comprising two bidirectional LSTM layers (**128 hidden units each**) with dropout regularization (**rate** = 0.2) to mitigate overfitting. Input sequences were generated by concatenating all 48 selected variables into free-text narratives, tokenized and padded to a uniform length of 512 tokens. The model utilized the **Adam** optimizer (**learning rate** = 0.001, β₁ = 0.9, β₂ = 0.999) with gradient clipping (**threshold** = 1.0) to stabilize training. Hyperparameter optimization was performed via grid search combined with 5-fold cross-validation, exploring variations in **hidden layer dimensions** (64, 128, 256 units), **batch sizes** (32, 64, 128), and learning rate schedules (step decay vs. exponential decay). **Early stopping** was applied with a patience of 10 epochs to halt training if validation loss plateaued. To enhance generalization, **L2** regularization (λ = 0.01) was incorporated into the fully connected output layer (sigmoid activation).
